# RETRACTION: Exploring the prophylactic role of soy isoflavones against polycystic ovarian syndrome

**DOI:** 10.1002/fsn3.4173

**Published:** 2024-05-02

## Abstract

**RETRACTION:** Nishaf Manzar, Safwan Ahmad khan, Najum Fatima, Mehr un Nisa, Muhammad Haseeb Ahmad, Muhammad Inam Afzal, Hafiz Fahad Ullah Saeed, Muhammad Imran, Faqir Muhammad Anjum, and Muhammad Sajid Arshad, “Exploring the prophylactic role of soy isoflavones against polycystic ovarian syndrome,” *Food Science & Nutrition* *9* (2021): 4738–4744. https://doi.org/10.1002/fsn3.2322.

The above article, published online on 09 July 2021 in Wiley Online Library (wileyonlinelibrary.com), has been retracted by agreement between the journal's Editor‐in‐Chief, Y. Martin Lo, and Wiley Periodicals, LLC. The retraction has been agreed to following concerns about the peer review process. The editorial office found unambiguous evidence that the manuscript was accepted solely based on compromised and insufficient reviewer reports. This evidence was further investigated and verified by the publisher and the Editor‐in‐Chief. As a result, the conclusions of this article are not considered reliable. The authors were notified of the retraction and disagreed with this decision.

**RETRACTION:** Nishaf Manzar, Safwan Ahmad khan, Najum Fatima, Mehr un Nisa, Muhammad Haseeb Ahmad, Muhammad Inam Afzal, Hafiz Fahad Ullah Saeed, Muhammad Imran, Faqir Muhammad Anjum, and Muhammad Sajid Arshad, “Exploring the prophylactic role of soy isoflavones against polycystic ovarian syndrome,” *Food Science & Nutrition* *9* (2021): 4738–4744. https://doi.org/10.1002/fsn3.2322.

The above article, published online on 09 July 2021 in Wiley Online Library (wileyonlinelibrary.com), has been retracted by agreement between the journal's Editor‐in‐Chief, Y. Martin Lo, and Wiley Periodicals, LLC. The retraction has been agreed to following concerns about the peer review process. The editorial office found unambiguous evidence that the manuscript was accepted solely based on compromised and insufficient reviewer reports. This evidence was further investigated and verified by the publisher and the Editor‐in‐Chief. As a result, the conclusions of this article are not considered reliable. The authors were notified of the retraction and disagreed with this decision.

